# Trials and Tribulations with VH Replacement

**DOI:** 10.3389/fimmu.2014.00010

**Published:** 2014-01-30

**Authors:** Wenzhao Meng, Sahana Jayaraman, Bochao Zhang, Gregory W. Schwartz, Robert D. Daber, Uri Hershberg, Alfred L. Garfall, Christopher S. Carlson, Eline T. Luning Prak

**Affiliations:** ^1^Department of Pathology and Laboratory Medicine, Perelman School of Medicine, University of Pennsylvania, Philadelphia, PA, USA; ^2^School of Biomedical Engineering, Science and Health Systems, Drexel University, Philadelphia, PA, USA; ^3^Center for Personalized Diagnostics, University of Pennsylvania Health System, Philadelphia, PA, USA; ^4^Department of Microbiology and Immunology, College of Medicine, Drexel University, Philadelphia, PA, USA; ^5^Division of Hematology-Oncology, Department of Medicine, Perelman School of Medicine, University of Pennsylvania, Philadelphia, PA, USA; ^6^Public Health Sciences Division, Fred Hutchinson Cancer Research Center, Seattle, WA, USA

**Keywords:** V(D)J recombination, VH replacement, VH, DH, and JH gene segments, receptor editing

## Abstract

VH replacement (VHR) is a type of antibody gene rearrangement in which an upstream heavy chain variable gene segment (VH) invades a pre-existing rearrangement (VDJ). In this Hypothesis and Theory article, we begin by reviewing the mechanism of VHR, its developmental timing and its potential biological consequences. Then we explore the hypothesis that specific sequence motifs called footprints reflect VHR versus other processes. We provide a compilation of footprint sequences from different regions of the antibody heavy chain, and include data from the literature and from a high throughput sequencing experiment to evaluate the significance of footprint sequences. We conclude by discussing the difficulties of attributing footprints to VHR.

## Context, Definition, and Potential Mechanisms of VH Replacement

Antibodies are heterotetrameric proteins comprised of two heavy chains and two light chains that are formed through V(D)J recombination to generate a highly diverse repertoire of antigen binding receptors expressed by B cells. The *recombinase activating gene* encoded proteins, RAG1 and RAG2, target conserved heptamer and nonamers within recombination signal sequences (RSSs) to cleave the DNA that flanks recombining gene segments that join together to form the variable regions of antibody heavy and light chains [reviewed in Ref. (1)]. Typical V(D)J recombination generates a signal joint and a coding joint, and the latter is further diversified at the junction between the recombining gene segments by mechanisms including P-addition, N-addition, and exonucleolytic nibbling [reviewed in Ref. (2)]. Occasionally atypical rearrangements occur, generating hybrid joints, open-and-shut joints, or joints between RSSs that ordinarily do not recombine (2–5).

Antibodies can be further revised and diversified through receptor editing of the light chain, somatic hypermutation, gene conversion, and VH replacement (VHR). Receptor editing typically involves RAG-dependent leapfrogging rearrangements on the same allele as the defective or autoreactive light chain, rearrangement on other alleles (κ or λ) and/or RS deletion [which renders preceding κ rearrangement non-functional, reviewed in Ref. (6)]. Somatic hypermutation is DNA point hypermutation carried out by activation induced cytidine deaminase (AID) (7), and typically signifies a T-cell dependent antibody response. Gene conversion, in which homologous sequences from other V genes are grafted into the functional V gene, is a common method of gene diversification in chickens (8), rabbits and more recent examples have been described in horses and humans (9), and appear to be AID-dependent (10). The final category of antibody gene diversification is VHR, which is the focus of this article. Replacement involves the transfer (or invasion) of some or most of another V gene into an existing gene rearrangement.

Darlow and Stott have reviewed the literature on VHR and envision two broad mechanistic classes of V replacement (11). The first, also termed “classical” VHR, consists of invasion of an existing VDJ rearrangement by an upstream VH. In classical VHR there is RAG-mediated cleavage at a cryptic RSS (cRSS) located in the 3′ end of the previously rearranged VH gene. The cRSS has a DNA sequence that differs from the conventional heptamer that flanks the DH gene segment by one nucleotide, bolded in the sequence that follows: 5′-**T**ACTGTG-3′ (12) and is found in ~70% of murine VHs and over 90% of human VHs (13). Occasionally other heptamers containing the 3′ GTG nucleotides can be used, suggesting that the last three nucleotides of the cRSS motif are critical (14, 15). The TGT within the cRSS is the codon encoding the conserved cysteine at the junction between FR3 and CDR3. The second class of replacement, according to Darlow and Stott, involves the transfer of other sequences of homology between different V genes at different sites, many of which appear to also resemble cRSSs. Examples of this second category of VHR have been described in antibodies cloned from single B cells in human tonsils (16), in antibodies cloned from synovial tissue of patients with rheumatoid arthritis (17), and in antibodies cloned from human mucosa associated lymphoid tissue lymphomas (18). Alternatively or in addition to RAG-mediated rearrangement, replacements in this second category may arise due to AID-mediated homologous recombination events that are unrelated to the putative cRSSs (11). However, the mechanism of type 2 replacement is far from resolved as recently a non-AID-dependent form of replacement has been described at the κ locus using human pre-B cell lines (19). As the molecular mechanism of type 2 replacement remains to be fully elucidated, we will focus the remainder of our analysis in this manuscript on classical VHR (which we refer to hereafter as “VH replacement”).

**Figure 1:**
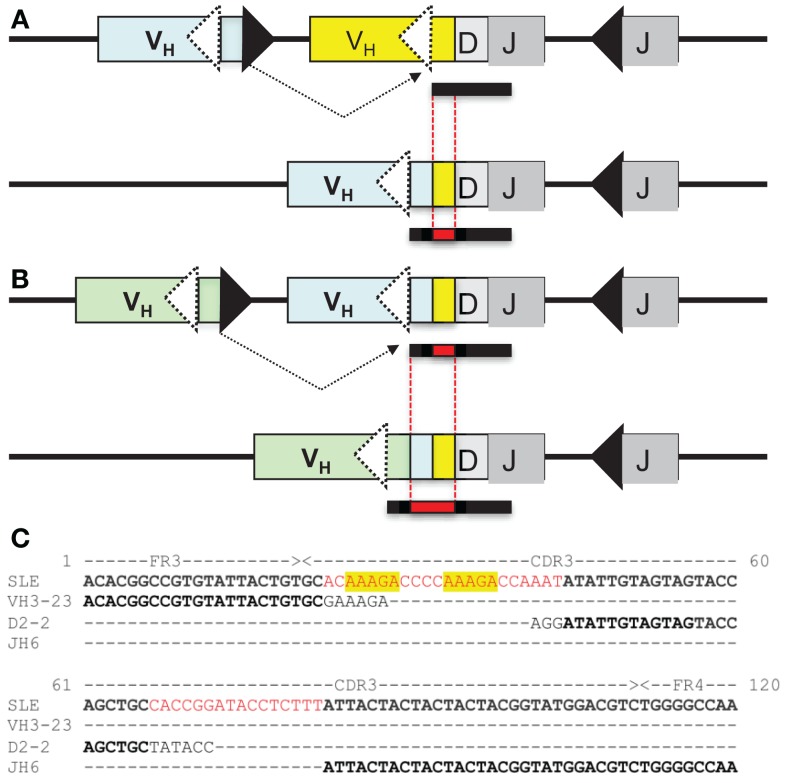
**(A)** VH replacement: an upstream VH gene invades by rearranging into a pre-existing rearrangement. RAG cleaves the conventional recombination signal sequence (black triangle) of the invading VH (light blue VH gene) and cleaves at a cryptic heptamer sequence (cRSS, dashed white triangle) of the invaded VH gene (yellow VH gene). The resulting rearrangement is shown on the second line of the diagram and includes the DH and JH genes of the previous rearrangement and the new VH gene. Also included in the VH replacement product is a remnant or “footprint” of the preceding VH gene (denoted by a yellow box). Often the products of VH replacement exhibit CDR3 elongation, due to the retention of the footprint sequence (the CDR3 is indicated by the bar under the sequence and the added length of the new CDR3 sequence including the footprint (in red) is indicated by the black bar below the sequence.) **(B)** Serial VH replacement. The same conventions are used as in **(A)** and a longer CDR3 is generated, via the accumulation of footprint sequences. In both panels, boxes denote exons, lines introns, triangles RSS and the rearrangement is indicated by dashed black lines. This diagram is not drawn to scale. **(C)** Long CDR3 sequence with possible VH replacement(s). Shown is the nucleotide sequence of an expanded B cell clone that was recovered from peripheral blood DNA of a patient with systemic lupus erythematosus (SLE) that reveals a 91 nucleotide CDR3. Kowal et al. described an anti-dsDNA H chain sequence comprised of a VH3-N-DH2-2-JH6, which has similar features to this junction, although it was shorter (21). Sequences in black font match the corresponding germline gene segments. Red font denotes possible N-additions and yellow shading highlights potential footprint sequences. Dashes indicate regions where sequences do not overlap. FR3, framework region 3; CDR3, third complementarity determining sequence; FR4, framework region 4.

During VHR, an upstream VH gene invades into the cRSS, replacing all but the last few nucleotides of the previously rearranged VH gene (Figure 1A). The remaining 3′ nucleotides of the VH, DH, and JH gene segments are retained in the new rearrangement. The extra nucleotides from the 3′ end of the previous VH gene are sometimes referred to as a “footprint.” Nearly all human VH genes have between five and nine nucleotides in the potential footprint, between the cRSS and the RSS. Most primary RSS rearrangements delete several of these nucleotides from the 3′ end, so the potential footprint may not be easily recognizable. Moreover, during VHR, additional nucleotides can be deleted, so the footprint from the primary VH can be entirely lost during VHR. It is also possible for more than one replacement rearrangement to occur on the same heavy chain allele, a process referred to as “serial” or “successive” VHR (Figure 1B) (20). An example of a heavy chain rearrangement with more than one footprint sequence is given in Figure 1C.

## Demonstration of VH Replacement in Cell Lines and Mouse Models

VH replacement was initially discovered in two different transformed B cell lines (12, 22). In both of these early studies, B cells with non-functional heavy chain gene rearrangements (VDJ−) were able to generate functional heavy chains (VDJ+) by undergoing further heavy chain rearrangement into the cRSS. Continued VHR could also convert a functional VDJ+ rearrangement into a non-functional one through the incorporation of an upstream pseudo-VH gene (12).

The development of antibody heavy chain (IgH) knock-in mice provided a formal demonstration of VHR in B cells *in vivo*. VHR was documented in hybridomas derived from the 3H9 heavy chain knock-in mouse (13). VHR and invasion of upstream DH gene also occurred in a knock-in for the T15 heavy chain (15). Furthermore, B cells from quasi-monoclonal mice, which have an anti-(4-hydroxy-3-nitrophenyl) acetyl (NP) heavy chain knock-in and can only produce λ light chains, due to homozygous engineered κ deficiency, can lose reactivity to NP by VHR. Strikingly, most secreted antibodies in the quasi-monoclonal mouse appear to arise through VHR (23). VHR was also observed in mice that were genetically engineered to contain two non-productively rearranged heavy chain alleles. In these VDJ−/VDJ− mice, IgHs were generated via VHR in a RAG-dependent manner (crossing the VDJ−/VDJ− mice onto a RAG2 deficient background resulted in a failure to generate IgM+ B cells) (24). The ability of RAG1 and RAG2 to bind to the cRSS was also demonstrated by electrophoretic mobility shift assays using VH4-34 cRSS versus consensus 12-RSS sequences (25).

In all of the preceding mouse models, VHR conferred greater diversity or functionality upon the B cell repertoire (i.e., there was a selective pressure that favored VHR). In contrast, when VHR was compared with conventional rearrangement, using a mouse model with an out of frame VDJ rearrangement (VDJ−) that was knocked into the heavy chain locus, conventional rearrangement on the other heavy chain allele occurred far more frequently (26). Similarly, in the 56R anti-dsDNA heavy chain knock-in mouse, receptor editing was far more efficient in B cells that were heterozygous rather than homozygous for 56R (27). One caveat to the 56R study was that cells that had undergone VHR on one allele but were still left with a functional copy of the DNA-reactive 56R heavy chain on the other allele could be counter-selected.

## VH Replacement in Bone Marrow B Cells

To gain further insight into the mechanism of VHR, studies were performed in mice to determine its developmental timing. Several studies suggest that VHR occurs at or near the time of conventional IgH gene rearrangement. The junctions of IgH sequences with evidence of VHR in IgH knock-in mice usually contain N-additions (13). Terminal deoxynucleotidyl transferase (TdT), the enzyme that carries out N-addition, is typically expressed at highest levels during H chain rearrangement in pro-B and large cycling pre-B cells (28). Therefore, the presence of N-additions provides indirect evidence that VHR occurred at the time when TdT was active and therefore probably took place in pro-B or early pre-B cells. Further evidence in support of VHR in early stage B cells includes ligation-mediated PCR to measure DNA breaks at the heavy chain locus, which occurred at the highest levels in pro-B cells (29). These studies suggest that VHR is either occurring in cells where IgH rearrangement has not yet shut down (failed allelic exclusion) or is driven by pre-BCR rather than BCR signaling, since only the former receptor is expressed at the pre-B cell stage of development.

With respect to pre-BCR signaling [reviewed in Ref. (30)], it is noteworthy that surrogate light chain knock-out mice have autoreactive antibodies with long CDR3 sequences (31). One potential explanation for this result is that, in the absence of surrogate light chain, the pre-BCR does not assemble and turn off heavy chain rearrangement. Without a heavy chain rearrangement stop signal, there may be higher frequencies of VHR, leading to CDR3 elongation. However, an alternative possibility is that peripheral selection of B cells with long CDR3 sequences is relaxed in the lymphopenic setting that arises due to inefficient primary B cell production in surrogate light chain knock-out mice. It is known that in the absence of normal numbers of peripheral B cells, the level of the B cell survival factor BLyS (also known as BAFF) increases, since B cells are the primary consumers of BLyS. It is also known that the stringency of B cell selection can be reduced when BLyS levels are increased (32, 33).

## VH Replacement in Peripheral B Cells

Some studies suggest that VHR could occur in more mature B cell subsets. For example, there are data implicating BCR signaling in VHR in the EU12 human B cell line, which phenotypically resembles IgM+, CD10+, CD24^high^ cells. In these cells, BCR crosslinking promotes VHR and, conversely, Syk and Src kinase inhibitors inhibit VHR (34). While some of the kinase inhibition experiments could also be influencing mechanisms that operate at earlier stages of B cell development, the BCR crosslinking experiment suggests that BCR signaling could promote VHR in more mature B cells. Furthermore, ligation-mediated PCR experiments documented double-stranded DNA breaks at VH3 cRSS sites in human immature (IgM+, CD27−, CD10+) and mature naïve (IgM+, CD27−, CD10−) circulating B cells, also suggesting that VHR may not be limited to immature B cells (34).

Chronic graft versus host disease (GVHD) is one of the most intriguing examples in which VHR could be occurring in more mature B cells (35). B6 mice injected with I–A incompatible T cells from bm12 mice develop chronic GVHD and produce a spectrum of autoantibodies that resembles those found in systemic autoimmune conditions such as systemic lupus erythematosus (SLE) (36). When anti-dsDNA heavy chain knock-in mice such as 3H9 and 56R are used, GVHD occurs and the production of anti-nuclear antibodies is enhanced (35). But the remarkable finding is that among IgG antibodies, a large fraction does not use the knocked in heavy chain (35). Although this unexpected skewing away from the 56R H chain could be the result of selective pressures on the minority population of H chain edited B cells that emerge from the bone marrow, it is not at all obvious how this selection could operate to disfavor the transgene, and why its effects would be largely confined to IgG and not IgM. It is possible that the transgene was revised (by further gene rearrangement) in the periphery, either because it was inactivated by somatic mutation (37), or because the stimulus afforded by cGVH re-induced the rearrangement machinery. An alternative explanation is that the 56R transgene bearing cells are disfavored during primary B cell maturation because they recognize DNA and this self-reactivity causes them to be anergized (this would predict that 56R+ cells would be over-represented amongst IgM rather than IgG B cells). Consistent with the possibility of anergy, most B cells expressing the IgM allotype of the 56R transgene have low levels of IgM (38–40).

## What are the Consequences and Potential Functions of VH Replacement?

VH replacement allows a B cell with an inadequate pre-BCR or an autoreactive BCR to swap out the existing heavy chain and replace it with a different heavy chain. But why would this be useful? One possibility is that VHR increases the odds of generating a functional antibody. Producing a functional antibody is rather difficult (41): many rearrangements are out of frame, VH pseudogenes outnumber functional VH genes, many newly generated antibodies are autoreactive (42), some combinations may be sequestered inside the cells (38) and some H and L chain combinations may not pair well with each other. VHR may also facilitate the use of a wider array of upstream VH genes. By giving cells with defective antibody rearrangements a chance at revising those antibodies, perhaps the efficiency of primary B cell generation is greatly improved.

**Figure 2:**
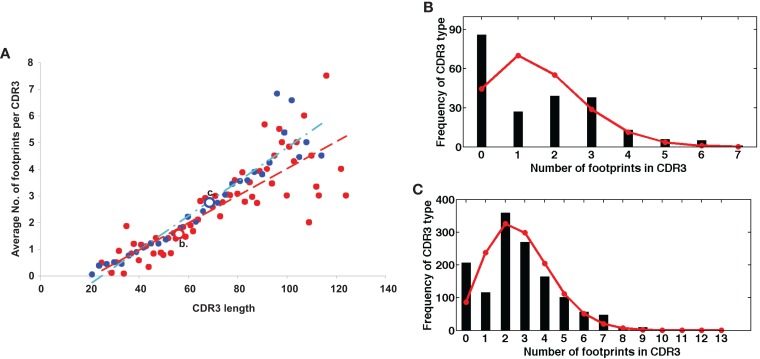
**(A)** Longer CDR3 sequences have more footprints. Plotted are the average numbers of footprint 5-mers per sequence. Sequences are averaged at each CDR3 size by the number of sequences that have CDR3s of that particular length. Blue dots are in-frame (IF) rearrangements and red dots are out of frame (OF) rearrangements. We find a positive linear correlation between the length of the CDR3 and the number of footprints (*r*^2^ = 0.89 for IF, *r*^2^ = 0.7 for OF). The line describing this relationship has a slope of 0.06 ± 0.008 for OF (red line) and 0.05 ± 0.008 for IF rearrangements (blue line). **(B,C)** are two examples of the entire distribution of footprint numbers at two positions – position 56 (red circle OF) and position 69 (blue circle IF). Black stems indicate the numbers of footprints and red lines represent the fit of a Poisson distribution (λ = 1.57 and 2.74, respectively).

On the other hand, a seemingly diametrically opposed consequence of VHR is the potential generation of multireactive antibodies. VHR can sometimes result in the retention of a “footprint” that is comprised of DNA sequences downstream of the cryptic heptamer of the invaded VH gene (Figure 1). Because the cRSS is typically positioned further from the WGXG motif in the JH segment than the 5′RSS of a DH segment, VHR is likely to produce longer CDR3 segments than primary rearrangements. Not surprisingly, longer CDR3 sequences have a higher proportion of footprints (Figure 2), but this does not guarantee that all long CDR3 are the product of VHR. Seventy-eight percent of the potential footprint regions in functional human VH genes contain an arginine codon, so footprint-containing sequences often also harbor a larger number of charged residues. Longer CDR3s have been associated with greater multireactivity, and such multireactive B cells are normally counter-selected as B cells mature during normal B cell development (42). RA patients have antibodies with unusual CDR3 sequences in their synovium (17) and we have seen CDR3 sequences in patients with SLE that have regions of sequence homology that could arise due to VHR. For example, Figure 1C shows a rearrangement from an expanded B cell clone in a patient with SLE that appears to contain two footprint sequences (highlighted in yellow). Autoimmune-prone strains of mice have elongated CDR3s, although many of these may arise through mechanisms other than VHR, such as D–D fusion (43, 44). All of these findings beg the question of whether such “multireactivity” serves a useful function. Is multireactivity protective, particularly in the context of an innate immune response? Or could multireactive antibodies be useful in clearing debris that might be inflammatory if left to accumulate? It is intriguing in this regard that some multireactive IgM antibodies such as the famous T15 idiotype, which binds phosphorylcholine (45), also have anti-inflammatory properties (46).

It is possible that there is no simple single answer to the function of VHR, if it has one at all. It would certainly seem that the biological consequences of VHR depend upon the developmental context in which the rearrangement occurs. If replacement occurs centrally, as is likely to occur in wild type strains of mice such as B6 (40, 47), it could serve as a tolerance mechanism (receptor editing) or as means of increasing the efficiency of primary B cell generation. It might also generate a portion of the primary antibody repertoire that has special functional properties such as multireactivity. Conversely, if it occurs peripherally, as might arise in dysregulated states of immune activation such as GVHD (48), perhaps autoimmunity results.

## VH Replacement in pre-B Cell ALL

Given the abundance of findings linking VHR to pro- or pre-B cell development discussed above, it is not surprising that the initial demonstrations of VHR occurred in transformed pre-B cell lines. More recently, VHR has been demonstrated to be a major contributor to clonal evolution in precursor B cell acute lymphoblastic leukemia (B-ALL) (49, 50). In B-ALL, there is presumably a large clone of cells “frozen” in the pre-B cell stage. The recombinase machinery remains active in at least some of these cells and can drive VHR. It is instructive to review the early work in the murine pre-B cell line NFS5, in which VHR was found to alter not only the productive but also the non-productively rearranged allele (12). Thus assays where one attempts to define a clone based upon its predicted “conservation” of other immunoglobulin gene rearrangements (such as the other H chain allele) within the same cell are not necessarily reliable or easy to interpret. The potential for VHR to contribute to intraclonal diversification is highly relevant to the design and interpretation of assays for minimal residual disease monitoring that employ quantitative PCR with probes or primers for clone-specific junctional sequences (51) or, more recently, high throughput sequencing of heavy chain CDR3 (52). Such studies must take VHR and other forms of intraclonal diversification into account.

## Analysis of VH Replacement Footprints

The most convincing demonstrations of VHR are those in which a precursor–product relationship can be documented. For example, if the precursor VH gene is known and then additional B cells can be found to share most of the 3′ side of the CDR3 (the same DH–JH junction), but have a different VH gene, this can be compelling, as in B-ALL or in mouse models with heavy chain knock-ins. In contrast, the analysis of VHR in a physiologic and fully diversified immune repertoire has by necessity focused on indirect evidence, namely the enumeration of footprints, which are potential traces of previous VDJ rearrangements in IgH sequence data. In mice, footprints are readily observed in constrained immune repertoires [for example, Ref. (13, 15, 23)]. Footprints are also observed in humans (53). However, a fundamental issue with footprint analysis in humans is one of specificity of attribution: does the footprint arise due to VHR or is it due to some other form of junctional diversification or skewing in the rearrangement process? Or does it occur by chance?

To investigate the hypothesis that footprint sequences are due to the process of VHR, we sequenced IgH rearrangements from peripheral blood B cells of a healthy human adult subject, following an IRB-approved protocol. We identified 42,221 unique sequences from this sample, which we analyzed for VH footprints using a sliding window method (see Supplementary Material for further details). All of the potential footprints arising from sequences at the 3′ ends of the germline VH genes are listed in Table 1. In accordance with their conventional description in the literature (41), we required the footprint to be least five nucleotides long (we hereafter refer to these sequences as footprint 5-mers). If footprint 5-mers are due to VHR, they will have specific characteristics in antibody repertoire data, indicated by the tests described below.

**Table 1:** **Footprint sequences in the 3′ end of human germline VH genes and alleles**.

Footprint (5-mer variants)	VH gene allele(s)	
CGAGAGA (CGAGA, GAGAG, AGAGA)	CGAGAGA	VH1-18, VH1-2*1, VH1-2*2, VH1-2*3, VH1-2*5, VH1-3, VH1-46*1, VH1-46*2, VH1-69*1, VH1-69*4, VH1-69*6, VH1-69*8, VH1-69*9, VH1-69*10, VH1-69*11, VH1-69*12, VH1-69*13, VH1/OR15-1*2, VH1/OR15-1*3, VH1/OR15-1*4, VH3-11*1, VH3-11*4, VH3-11*5, VH3-21, VH3-30*1, VH3-30*3, VH3-30*4, VH3-30*5, VH3-30*6, VH3-30*7, VH3-30*9, VH3-30*10, VH3-30*11, VH3-30*12, VH3-30*13, VH3-30*14, VH3-30*15, VH3-30*16, VH3-30*17, VH3-30*18, VH3-30*19, VH3-33*1, VH3-33*2, VH3-33*4, VH3-33*5, VH3-48, VH3-53*1, VH3-53*4, VH3-64*1, VH3-64*2, VH3-64*4, VH3-66*1, VH3-66*3, VH3-7*1, VH3-7*3, VH4-28*3, VH4-30-2*4, VH4-31*1, VH4-31*2, VH4-31*3, VH4-31*10, VH4-34*9, VH4-39*2, VH4-39*6, VH4-39*7, VH4-4*2, VH4-4*6, VH4-4*7, VH4-59*1, VH4-59*2, VH4-61*1, VH4-61*2, VH4-61*3, VH4-61*8, VH4/OR15-8, VH7-4-1*2, VH7-4-1*4, VH7-4-1*5
	CGAGA	VH1-2*4, VH1-69*2, VH1-69*5, VH1/OR15-1*1, VH3-11*3, VH3-30*8, VH3-30-3*1, VH3-53*2, VH3-66*2, VH3-7*2, VH4-28*4, VH4-34*12, VH4-59*7, VH4-61*5, VH4-b, VH5-51*3, VH5-51*4, VH5-a, VH7-4-1*1
CGAGAGG (CGAGA, AGAGG)	VH1-8, VH4-34*1, VH4-34*2, VH4-34*4, VH4-34*5, VH4-34*13, VH4-59*9	
CGAGACA (CGAGA, GAGAC, AGACA)	VH3-66*4, VH4-30-2*3, VH4-39*1, VH4-59*8, VH4-61*7, VH5-51*1, VH5-51*2	
CGAGATA (CGAGA, GAGAT, AGATA)	VH4-34*10, VH4-59*10, VH7-81	
CGAGAAA (CGAGA, GAGAA, AGAAA)	VH4-28*1, VH4-28*2, VH4-28*5, VH4-28*6	
CAAGA*N*A (CAAGA, *AAGAN, AGANA*)	*CAAGANA*	VH1-45*1
	CAAGATA	VH1-45*2
	CAAGAGA	VH3-13*1, VH3-13*2, VH3-13*4, VH3-74*1, VH3-74*3, VH3/OR16-10*3, VH6-1
	CAAGA	VH1-45*3, VH3-13*3, VH3-74*2, VH3/OR16-10*1, VH3/OR16-10*2, VH3/OR16-12
CAACAGA	CAACAGA	VH1-24
	CAACA	VH1-f*1
CTAGAGA (CTAGA, TAGAG, AGAGA)	CTAGAGA	VH1-46*3, VH3-72*1, VH3/OR15-7*5
	CTAGA	VH3/OR15-7*1, VH3/OR15-7*2, VH3/OR15-7*3
CTAGGGA (CTAGG, TAGGG, 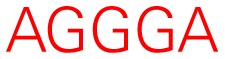 )	VH3-53*3
CGAAAGA (CGAAA, GAAAG, AAAGA)	CGAAAGA	VH3-23*1, VH3-23*2, VH3-23*4, VH3-30*2, VH3-30-3*2, VH3-33*3, VH3-33*6, VH3-NL1
	CGAAA	VH3-23*3, VH3-23*5
	*CGNNN*	VH4-30-2*2, VH4-31*4, VH4-34*8, VH4-39*5, VH4-59*3, VH4-59*4, VH4-59*5, VH4-59*6,
	*CG*	VH4-31*5
CCAGATATA (CCAGA, CAGAT, AGATA, 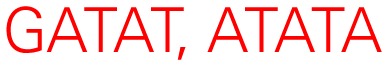 )	VH3-38
CCAGAGA (CCAGA, CAGAG, AGAGA)	VH4-30-2*1, VH4-30-2*5, VH4-30-4*1, VH4-30-4*2, VH4-30-4*5, VH4-30-4*6, VH4-61*6	
TGAAACA (TGAAA, GAAAC, AAACA)	TGAAA	VH3/OR16-8*1, VH3/OR16-9
	TGAAACA	VH3/OR16-8*2
TGAGA		
TGAGAGA (TGAGA, GAGAG, AGAGA)	TGAGA	VH1/OR15-5
	TGAGAGA	VH1/OR15-9, VH1/OR21-1
TGAGAAA (TGAGA, GAGAA, AGAAA)	VH3-16, VH3-35
TGAAAGA (TGAAA, GAAAG, AAAGA)	VH3-64*3, VH3-64*5	
CGGCAGA (CGGCA, GGCAG, GCAGA)	VH1-58
CACGGATAC (CACGG, ACGGA, CGGAT, 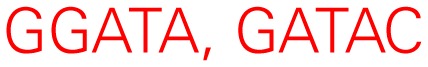 )	VH2-26, VH2-70*1, VH2-70*10, VH2-70*11	
CATGGAGAG ( 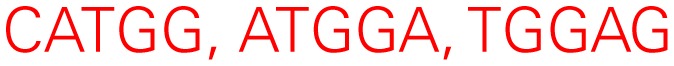 , GGAGA, GAGAG)	VH2/OR16-5
TACGG	VH2-5*4, VH2-70*9
*TANNN*	VH2-5*7
CACGG	VH2-5*10
CACACAGACC (CACAC, ACACA, CACAG, ACAGA, CAGAC, AGACC)	CACACAGACC	VH2-5*1
	CACACAGAC	VH2-5*5, VH2-5*8, VH2-5*9, VH2-70*12
	CACACAGA	VH2-5*6
CAAAAGATA (CAAAA, AAAAG, AAAGA, AAGAT, AGATA)	VH3-43, VH3-9

Two hundred and seventy-three functional VH genes, including alleles and sequences designated as open reading frames, were downloaded from the IMGT database (54) and manually scanned for footprints. A footprint is defined by the nucleotide sequence following the cryptic recombination signal sequence (cRSS), TACTGTG, at the 3′ end of each VH, and is listed in the left column of the table. The footprint 5-mer variants that were used to scan the sequences are included in parentheses. The right column lists the VH genes and alleles. An asterisk in the VH name refers to a specific allele. If all alleles of a particular VH have the same footprint, allele names are omitted. Overlapping footprints are listed for some footprints in the sub-column on the right. Footprints in red font are also found in germline DH and JH gene segments. Sequences with ambiguous nucleotide designations (N-nucleotides) are indicated by italic font. The following VH gene alleles do not have a cRSS: VH1-18*2, VH1-69*3, VH1-69*7, VH1-c, VH1-f*2, VH2-5*2, VH2-5*3, VH2-70*2, VH2-70*3, VH2-70*4, VH2-70*5, VH2-70*6, VH2-70*7, VH2-70*8, VH2-70*13, VH3-15, VH3-20, VH3-25*4, VH3-49, VH3-72*2, VH3-73*1, VH3-73*2, VH3-d, VH3/OR16-13, VH3/OR16-6*2, VH4-30-4*3, VH4-30-4*4, VH4-31*6, VH4-31*7, VH4-31*8, VH4-31*9, VH4-34*3, VH4-34*6, VH4-34*7, VH4-34*11, VH4-39*3, VH4-39*4, VH4-4*1, VH4-4*3, VH4-4*4, VH4-4*5, VH4-61*4, VH5-51*5, VH7-4-1*3.

### Test 1: VH replacement footprints should be located in the 5′ end of the CDR3 sequence

**Figure 3:**
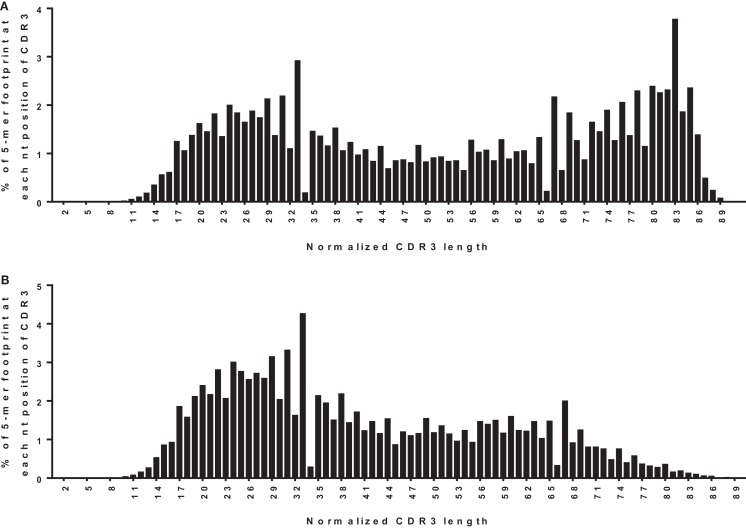
**Positions of footprint 5-mers**. **(A)** Frequency distribution of all footprint 5-mers out of total unique sequences (*n* = 42,221) plotted against the normalized CDR3 position. The CDR3 is herein defined to begin at the conserved CAR amino acid sequence (TGT GCG AGA nucleotide sequence) within the 3′ end of VH and end at the conserved W (TGG nucleotide sequence) that is immediately upstream of the first conserved glycine, GGC nucleotide sequence) within the JH. “TGGAG” is excluded as it is found in many alleles of all DH genes. The position of the footprint is defined by where the footprint starts within the CDR3. For example, if a footprint occupies nucleotides 12–16 of the CDR3, it will be plotted at position 12. CDR3 lengths were normalized to a scale of 1–100 using p1 = p/(L × 100); where p is the position of the 5-mer in the real CDR3 sequence, L is the length of the CDR3 sequence, and p1 is the normalized position. Normalized positions are rounded to the nearest integer. **(B)** Frequency distribution of all footprint 5-mers plotted against the normalized CDR3 position, corrected for footprint 5-mers in the germline JH6 gene. “ATGGA,” “TACGG,” and “CATGG” were excluded when found in the 3′ end of the CDR3 within the JH6 gene, as these 5-mers are found in the germline JH6 sequence. Footprint 5-mers found in other JHs (see Table 2) are not counted in either **(A)** or **(B)** because they are all located outside CDR3 region of JH.

One way to distinguish bona fide footprints from other sources of sequence variation is to compare the number of footprints in the junction between VH and DH (referred to as N1) to the number in the junction between DH and JH (referred to as N2). Footprints arising via VHR should occur in N1 rather than N2 because the cryptic heptamer is located in N1. However, as shown in Figure 3A, there is a roughly bimodal distribution of footprint 5-mers. Even though we excluded the most common footprints that were found in the germline DH gene segments (Table 2), there were still plenty of footprint sequences in N1, DH, and N2. In Figure 3B we took the analysis one step further and removed some more of the common footprint sequences that are found not only in the germline DH gene segments but also in JH6. This resulted in more skewing toward N1, but a large proportion of the footprints were still outside of N1. In fact, not only were footprint 5-mers found in DH and JH, but they were also found in other parts of the VH gene. Table 3 lists the positions of all of the footprint 5-mers found amongst the germline VH alleles listed in the IMGT database.

**Table 2:** **Footprint sequences in DH and JH alleles**.

DH gene	Sequence (footprint(s) in red font)
D1-1*01	GGTACAACTGGAACGAC
D1-14*01	GGTATAACCGGAACCAC
D1-20*01	GGTATAACTGGAACGAC
D1-26*01	GGTATAGTGGGAGCTACTAC
D1-7*01	GGTATAACTGGAACTAC
D2-15*01	A 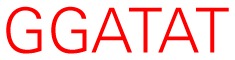 TGTAGTGGTGGTAGCTGCTACTCC
D2-2*01	A 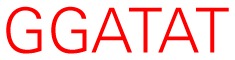 TGTAGTAGTACCAGCTGCTATGCC
D2-2*02	A 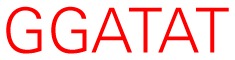 TGTAGTAGTACCAGCTGCTATACC
D2-2*03	T 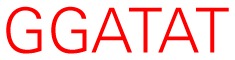 TGTAGTAGTACCAGCTGCTATGCC
D2-21*01	AGCATATTGTGGTGGTGATTGCTATTCC
D2-21*02	AGCATATTGTGGTGGTGACTGCTATTCC
D2-8*01	A 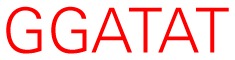 TGTACTAATGGTGTATGCTATACC
D2-8*02	A 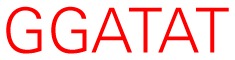 TGTACTGGTGGTGTATGCTATACC
D3-10*01	GTATTACTATGGTTCGGGGAGTTATTATAAC
D3-10*02	GTATTACTATGTTCGGGGAGTTATTATAAC
D3-16*01	GTATTATGATTACGTTTGGGGGAGTTATGCTTATACC
D3-16*02	GTATTATGATTACGTTTGGGGGAGTTATCGTTATACC
D3-22*01	GTATTACTATGATAGTAGTGGTTATTACTAC
D3-3*01	GTATTACGATTTT 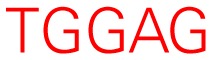 TGGTTATTATACC
D3-3*02	GTATTAGCATTTT 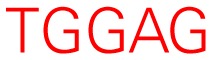 TGGTTATTATACC
D3-9*01	GTATTAC 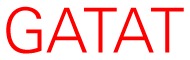 TTTGACTGGTTATTATAAC
D4-11*01	TGACTACAGTAACTAC
D4-17*01	TGAC 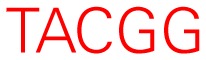 TGACTAC
D4-23*01	TGAC 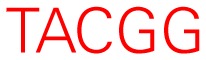 TGGTAACTCC
D4-4*01	TGACTACAGTAACTAC
D5-12*01	GT 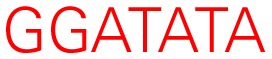 GTGGCTACGATTAC
D5-18*01	GT 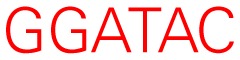 AGCTATGGTTAC
D5-24*01	G 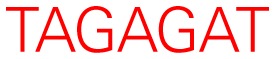 GGCTACAATTAC
D5-5*01	GT 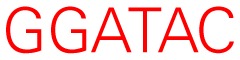 AGCTATGGTTAC
D6-13*01	GGGTATAGCAGCAGCTGGTAC
D6-19*01	GGGTATAGCAGTGGCTGGTAC
D6-25*01	GGGTATAGCAGCGGCTAC
D6-6*01	GAGTATAGCAGCTCGTCC
D7-27*01	CTAACTGGGGA

**JH gene**	**Sequence (footprint(s) in red font)**

J1*01	GCTGAATACTTCCAGCACTGGGGCCAGGGCACCCTGGTC ACCGTCTCCTCAG
J2*01	CTACTGGTACTTCGATCTCTGGGGCCGTGGCACCCTGGTC ACTGTCTCCTCAG
J3*01	TGATGCTTTTGATGTCTGGGGCCA 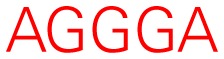 CAATGGTCACCG TCTCTTCAG
J3*02	TGATGCTTTTGATATCTGGGGCCA 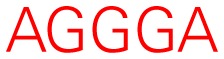 CAATGGTCACCG TCTCTTCAG
J4*01	ACTACTTTGACTACTGGGGCCAAGGAACCCTGGTCACCGTCT CCTCAG
J4*02	ACTACTTTGACTACTGGGGCC 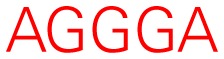 ACCCTGGTCACCGTCT CCTCAG
J4*03	GCTACTTTGACTACTGGGGCCA 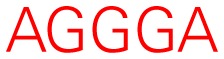 CCCTGGTCACCGTCT CCTCAG
J5*01	ACAACTGGTTCGACTCCTGGGGCCAAGGAACCCTGGTCACC GTCTCCTCAG
J5*02	ACAACTGGTTCGACCCCTGGGGCC 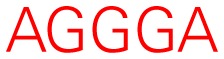 ACCCTGGTCACCGTCTCCTCAG
J6*01	ATTACTACTACTAC 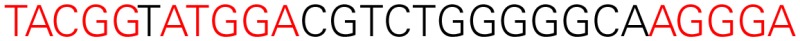 CCACGGTCACCGTCTCCTCAG
J6*02	ATTACTACTACTAC 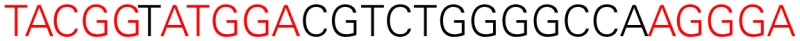 CCACGGTCACCGTCTCCTCAN
J6*03	ATTACTACTACTACTACTA 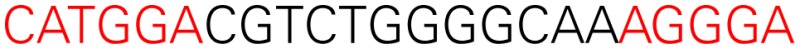 CCACGGTCACCGTCTCCTCAN
J6*04	ATTACTACTACTAC 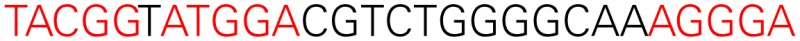 CCACGGTCACCGTCTCCTCAG

Thirty-four germline functional human DH alleles and 13 JH alleles were downloaded from the IMGT database (54). Each allele was scanned for all of the possible five nucleotide footprint motifs listed in Table 1. Sequences containing five nucleotide footprints are given in red font. In some cases the region matches more than one possible footprint (for example, D5-12*01 contains three different footprints: GGATA, GATAT, and ATATA).

**Table 3:** **The number of footprints found in various regions of human VH genes**.

Footprint	Sequences	FR	CDR	FR1	FR2	FR3	CDR1	CDR2	CDR3
TGAGA	131	212	12	94		118	3	3	6
CTAGAGA	8		8						8
CGAAAGA	9		9						9
CTAGA	28	17	16		17		1	1	14
TACGG	9	5	4	1		4	1	1	2
CAAAA	45	41	13			41		9	4
CTAGGGA	2	1	1		1				1
CAACA	55	33	27	1	5	27	2	23	2
CAAGA	150	149	18		4	145		3	15
CACACAGA	20	20	6	20					6
CGAGACA	7		7						7
CGAGAAA	3		3						3
TGAAAGA	2		2						2
TGAAACA	3		3					2	1
CGAGAGG	8	2	6		2				6
CGAGAGA	86		86						86
CACGG	179	178	6			178			6
CGAGATA	3		3						3
CGAAA	11		11					1	10
TGAAA	61	79	7	33		46	1	3	3
CACACAGAC	20	20	5	20					5
CGAGA	130	3	127	1	2				127
CACACAGACC	20	20	1	20					1
TGAGAAA	6		6					3	3
CAAAAGATA	3		3						3
CGGCAGA	2		2						2
TGAGAGA	3	1	2			1			2
CCAGATATA	2		2						2
CAAGATA	1		1						1
CCAGAGA	84	80	4			80			4
CACGGATAC	5		5						5
CAAGAGA	27	19	8			19			8

*Two hundred and thirty-four functional germline human VH alleles were downloaded from the IMGT database (54). For each footprint, the number of times that footprint was found in the VH alleles was recorded. The first column lists the footprint, the second column lists the number of alleles in which the footprint is found. Some alleles contain more than one footprint. The remaining columns indicate how many footprints were found in each of the corresponding regions of the V region. FR, framework; CDR, complementarity determining region. The columns named FR and CDR provide the total number of times that a particular footprint is found in the FR or the CDR*.

### Test 2: VH replacement footprints should be more frequent in upstream VH genes than downstream VH genes and absent from VH6-1

**Figure 4:**
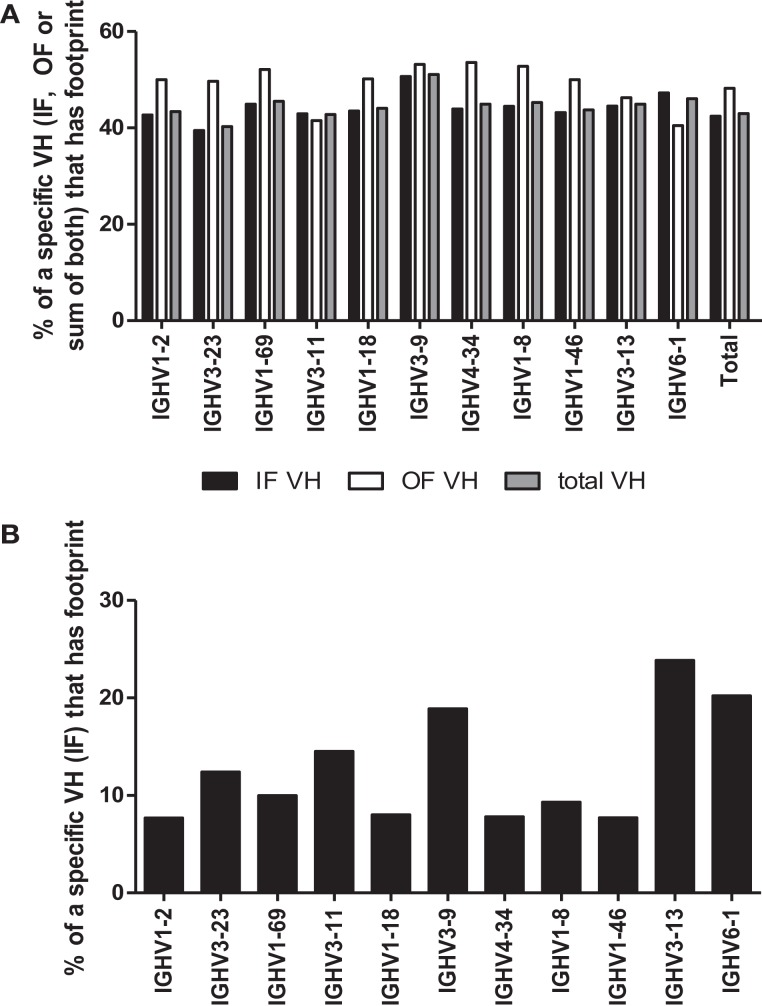
**(A)** Percentage of rearrangements with footprints for the 10 most frequent VH rearrangements and VH6-1. The percentages of rearrangements that contain at least one footprint 5-mer in either (or both) the 5′ end or the 3′ end of the rearrangement are shown for unique rearrangements for each of the 10 most common VH genes and for VH6-1. CDR3 sequences are defined as described in the legend to Figure 3 and normalized to an arbitrary length of 100. The 5′ end (which almost always contains N1) is defined as the first 20% of the sequence and the 3′ end is defined as the last 20% of the sequence (which almost always contains N2). VH6-1 is the most 3′ VH gene and cannot contain footprints that are due to VH replacement. Black bars denote unique rearrangements that are in-frame (IF VH), white bars denote out of frame rearrangements (OF VH), and gray bars indicate total unique rearrangements (total VH). **(B)** Footprint Frequency of in-Frame VH rearrangements in N1 using IgAT Software. The same unique in-frame (IF) VH rearrangements, as shown in **(A)**, were analyzed for the presence of one or more footprints using IgAT software (2). Plotted are the frequencies of unique IF VH rearrangements that have one or more footprints in the N1 region.

Another requirement for a footprint to be consistent with VHR is that the invading VH must be upstream of the VH that donated the footprint. Unfortunately, the recipient VH is often difficult to define because many VHs have the same or very similar footprints (see Table 1). However, a more straightforward test of whether a footprint 5-mer represents the product of VHR is to evaluate the frequency of footprints in different VH rearrangements. In particular, the 3′ most VH gene (VH6-1, in humans), when rearranged, should not exhibit VHR footprints as there is no downstream VH that it can invade. Conversely, VH genes that are situated in the 5′ end of the locus should have higher frequencies of footprints than 3′ VH genes, if VHR is frequent. Yet the overall frequency of footprint 5-mers was similar amongst unique sequences in all of the most commonly used VHs, including VH6-1 (Figure 4). The frequency of footprints was also not significantly higher in out of frame (unselected) versus in-frame rearrangements (Figure 4A).

We also performed this analysis using immunoglobulin analysis tool (IgAT) software (42) and observed that the frequency of footprints was not reduced in VH6-1 when compared to other VHs (Figure 4B). Lower VH footprint frequencies were observed overall because footprints in the 3′ end of the CDR3 are excluded by the IgAT program (42). One intriguing feature of the IgAT data was that, unlike our footprint analysis that captured 5-mers at both N1 and N2, when only N1 was analyzed, some VHs, including VH6-1, had higher footprint frequencies than others. Since VH6-1 cannot have any footprints due to VHR, we conclude that many footprint 5-mers that are found in the CDR3 do not arise by VHR.

The simplest explanation is that the great majority of 5-mer sequences found throughout the CDR3 resemble footprints by chance. The frequency of footprint 5-mers in the entire CDR3 was highly correlated with the length of the CDR3 (Figure 2). The ability to generate a replacement footprint by chance may be under-appreciated. In a completely random DNA sequence with equal proportions of A, T, G, and C bases, the chance of finding a specific 5-mer sequence is 1/1,024 (or ~0.001). However, there are at least 50 different footprint-derived 5-mer sequences amongst human VH genes (Table 3), increasing the odds to 50/1,024 (~5%). But this calculation ignores the number of different positions along the VDJ rearrangement where the footprint might be detected and on how many variants of the footprint are permitted. If the 5′ end of a CDR3 sequence is 30 nucleotides long, that means that there are 6 completely non-overlapping sequences that have a length of five nucleotides, bringing the minimum likelihood of detection of at least a single footprint in that sequence up to 26% [1−(1−0.05)^6^] or 1 − Pr(not getting any 5-mers in the 30 bp sequence). If the base composition of the DNA is non-uniform or the entire CDR3 sequence is surveyed or if sequences with mutations are permitted (for example those matching in 4 out of 5 bp), the chances of detecting a footprint increase even further.

We also wondered why some VH genes had higher footprint frequencies in N1 than others (Figure 4B), as this finding is not similar to what one would expect by random chance. We wondered if the real VHR events were hiding somewhere in a large pile of non-VHR footprints. A high “false positive” rate of footprint 5-mers could come about because of sequencing errors. Alternatively or in addition, it may be easy to create false VHR 5-mer sequences in primary VDJ rearrangements through a combination of N-addition, nibbling (or sequencing deletion) and the 3′ sequence of the VH. For example AAAGA could become AAGA or AGA.

It may be worthwhile to develop a better computational approach for detecting VHR footprints with greater specificity for VHR. The IgAT software already eliminated footprints that match the germline VH sequence exactly, but this is insufficient, give the high frequency of footprints in N1 of VH6-1. Further specificity might be achievable if one were to limit the detection of footprint 5-mers to sequences that are *unlikely* to arise through a single nucleotide change (for example, those that arise by deletion that converts a non-matching 6-mer to a matching 5-mer, or mutation of a non-matching 5-mer to a footprint 5-mer or N-addition of a nucleotide adjoining a 4-mer to create a footprint 5-mer). An alternative approach is to require that there be two footprint-like sequences in tandem. Either or both of these methods might increase specificity, but could also reduce sensitivity of footprint detection. The validity of either approach would need to be tested further using validated data sets in which VHR events are known to have or have not occurred.

We also considered the possibility that footprint 5-mers may frequently arise through some mechanism other than VHR. We considered two potential alternative mechanisms – (1) microhomology-mediated joining and (2) cleavage, nibbling, and rejoining at the cryptic heptamer.

### Alternative theory 1: Microhomology-mediated joining

**Figure 5:**
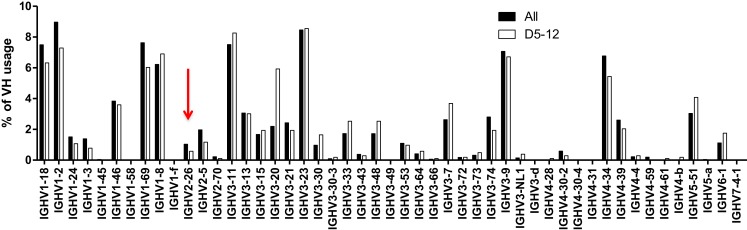
**VH usage is similar in all unique rearrangements and in rearrangements with DH5-12**. VH usage is shown for total unique sequences (closed bars, *n* = 42,221) and for those with DH5-12 (open bars, *n* = 1,029). Sequences containing the DH5-12 gene segment in the CDR3 region were recognized using IMGT high V quest analysis, and further analysis on VH usage was performed in-house (see text). The red arrow points to VH2-26, which contains some of the same footprint 5-mers as DH5-12, but does not appear to be used more frequently in rearrangements that include DH5-12.

We considered the possibility that footprints at N1 were arising primarily due to microhomology-mediated joining of similar sequences between the VH and the DH segments. If microhomology-mediated joining were common, one might expect that VHs that share the same 5-mers with DHs are more likely to rearrange, but as shown in Figure 5, this is probably not usually the case. DH5-12 (open bars in Figure 5), which has three footprint 5-mers, does not appear to be used more frequently in rearrangements involving VHs that contain the same 5-mers such as VH2-26 (red arrow). Rather than being skewed toward particular VHs with matching or similar footprints, the frequency of rearrangements of different VHs to DH5-12 rearrangements resembled overall VH usage (Figure 5, closed bars). While this analysis is very preliminary and only focused on a single DH, it suggests that microhomology-mediated joining, based upon shared sequences between VH and DH, is not a frequent mechanism for generating footprint 5-mers.

### Alternative theory 2: Cleavage, nibbling, and rejoining at the cryptic heptamer in VH

We wondered if there could be cleavage at the cryptic heptamer, followed by exonucleolytic nibbling and re-sealing at the site of the break, without full-blown rearrangement (Figure S1 in Supplementary Material illustrates this idea for a VH6-1 rearrangement). Note that this type of rearrangement product would not involve VHR, but would have the result of diversifying the 3′ end of the VH in the primary rearrangement product, altering the primary amino acid sequence and/or the reading frame of the rearrangement. This hypothesis makes predictions regarding the sequence characteristics that would be more or less amenable to this type of atypical open-and-shut joint (2). For example, one would expect that if most footprint 5-mers at N1 arise by this mechanism, that the frequency of footprint 5-mers would be very low in VH genes that lack cryptic heptamers. Furthermore, one would expect that the 5′ footprint 5-mer seen in most rearrangements would resemble the 3′ end of the germline sequence of the same VH gene that is present in the rearrangement.

### Preliminary conclusions and caveats from footprint analysis

Taken together, these data suggest that many if not most footprint sequences arise by some mechanism(s) other than VHR. But there are some caveats to this analysis. First, these data were only obtained on one healthy adult. It is possible that footprints may differ in other individuals or in a minority of individuals. In addition, different findings might occur in individuals with immunologic disorders such as SLE or neoplastic conditions such as B-ALL. Furthermore, only B cells from the peripheral blood were analyzed. It is conceivable that B cell populations with extensive VHR reside elsewhere in the body, particularly within the bone marrow. Finally, as discussed above, it is possible that some of the VHR footprints that were identified are due to sequencing errors. We tried to protect against this artifact by selectively analyzing unique sequences that were present in at least two copies. But even with this precaution, there are still likely to be many sequencing errors.

## Conclusion

VH replacement exchanges the VH within a pre-existing VDJ rearrangement with an upstream VH gene, while preserving most of the original CDR3 sequence. It also sometimes results in the retention of a footprint sequence in the VH gene that was invaded. The result of VHR is an alteration in the specificity or functional status of the antibody. But the mechanistic consequence of that alteration is unclear. Is it to diversify the repertoire once a good CDR3 sequence has been found? Or is it to reduce autoreactivity or generate some form of protective multireactivity? Or is it simply a means by which B cells with non-productive rearrangements on one or both alleles have another shot at creating a productive rearrangement? In humans, the analysis of VHR is confounded by not having a means of definitively identifying the precursor rearrangement. Rather, the analysis of VHR in humans is accomplished indirectly through footprint analysis, but as demonstrated herein, footprints may arise for reasons other than VHR. Thus, while VHR certainly occurs, footprint analysis is a poor measure of its frequency because of the high rate of false positives and an unknown rate of false negatives. Nevertheless, it is possible that footprints may provide other insights into the mechanisms of V(D)J recombination and its potentially aberrant regulation in disease states. With the advent of high throughput sequencing studies, further analysis of IgH gene rearrangements for VHR and other mechanisms of CDR3 diversification promise to be illuminating.

## Conflict of Interest Statement

Dr. Christopher S. Carlson is affiliated with Adaptive Biotechnologies, a for-profit high throughput sequencing company. The high throughput sequencing data that were analyzed for this paper were, however, generated at the University of Pennsylvania, not at Adaptive Biotechnologies. The other co-authors declare that the research was conducted in the absence of any commercial or financial relationships that could be construed as a potential conflict of interest.

## Supplementary Material

The Supplementary Material for this article can be found online at http://www.frontiersin.org/Journal/10.3389/fimmu.2014.00010/abstract

Figure S1 **Model for atypical open-and-shut joints**. Footprint 5-mers can be generated by cleaving at the cryptic heptamer, followed by preferential trimming by exonucleolytic nibbling at the 3′ end of the double strand break. Shown is the generation of a footprint sequence in a VH6-1 rearrangement. The cryptic heptamer is indicated by a dashed triangle, colored squares indicate the VH, DH, and JH gene segments (not drawn to scale). According to this model, there is cleavage at the cryptic heptamer, followed by nibbling at the 3′ end of the break (red wavy line), leading to selective loss of the A residue. The third line of the figure shows the repaired rearrangement, in which the A residue is missing from the rearrangement.

## Abbreviations

CDR3, third complementarity determining region; cRSS, cryptic recombination signal sequence; DH, antibody heavy chain diversity gene segment; footprint 5-mer, pentameric nucleotide sequence attributed to VHR; GVHD, graft versus host disease; IgH, antibody heavy chain; JH, antibody heavy chain joining gene segment; RSS, recombination signal sequence; SLE, systemic lupus erythematosus; VH, antibody heavy chain variable gene segment; VHR, VH replacement.
