# Supplementary material for: Trials and Tribulations with VH Replacement
Source: Front Immunol. 2014 Jan 30;5:10. doi: 10.3389/fimmu.2014.00010 (PMC3906580; doi:10.3389/fimmu.2014.00010)
Supplement: Figure S1 — Model for atypical open-and-shut joints. Footprint 5-mers can be generated by cleaving at the cryptic heptamer, followed by preferential trimming by exonucleolytic nibbling at the 3′ end of the double strand break. Shown is the generation of a footprint sequence in a VH6-1 rearrangement. The cryptic heptamer is indicated by a dashed triangle, colored squares indicate the VH, DH, and JH gene segments (not drawn to scale). According to this model, there is cleavage at the cryptic heptamer, followed by nibbling at the 3′ end of the break (red wavy line), leading to selective loss of the A residue. The third line of the figure shows the repaired rearrangement, in which the A residue is missing from the rearrangement. [file 73470_Luning_Prak_Presentation1.PPTX]

## Slide 1
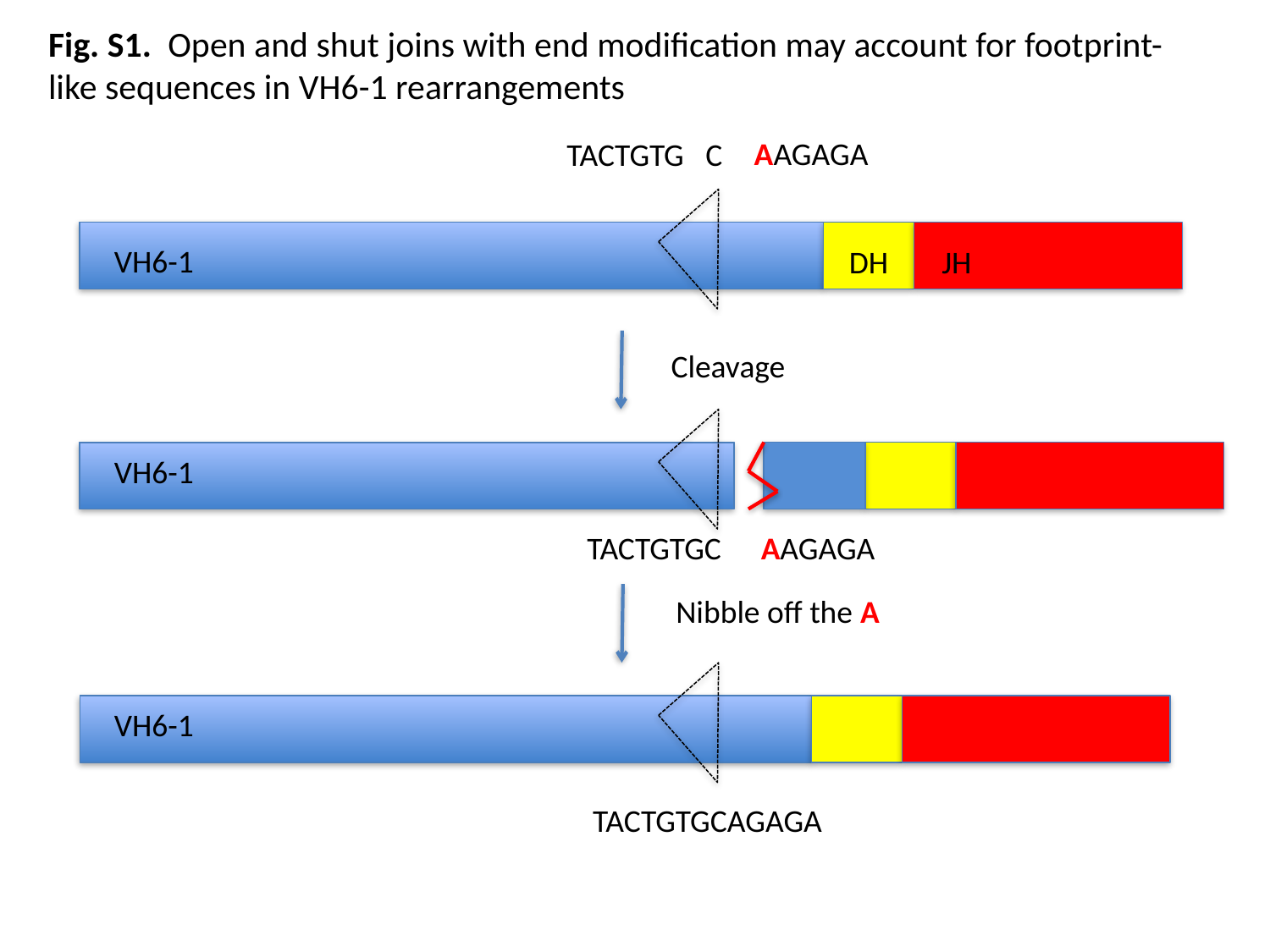

# Fig. S1. Open and shut joins with end modification may account for footprint-like sequences in VH6-1 rearrangements
AAGAGA
TACTGTG C
VH6-1
DH
JH
Cleavage
VH6-1
TACTGTGC
AAGAGA
Nibble off the A
VH6-1
TACTGTGCAGAGA
